# Recurrent reciprocal deletions and duplications of 16p13.11: the deletion is a risk factor for MR/MCA while the duplication may be a rare benign variant

**DOI:** 10.1136/jmg.2007.055202

**Published:** 2008-01-07

**Authors:** F D Hannes, A J Sharp, H C Mefford, T de Ravel, C A Ruivenkamp, M H Breuning, J-P Fryns, K Devriendt, G Van Buggenhout, A Vogels, H Stewart, R C Hennekam, G M Cooper, R Regan, S J L Knight, E E Eichler, J R Vermeesch

**Affiliations:** 1 Centre for Human Genetics, University Hospital, Catholic University of Leuven, Leuven, Belgium; 2 Department of Genome Sciences, University of Washington School of Medicine, Seattle, Washington, USA; 3 Department of Clinical Genetics, CHCG, Leiden University Medical Center, Leiden, The Netherlands; 4 Department of Clinical Genetics, Oxford Radcliffe Hospitals NHS Trust, Churchill Hospital, Oxford, UK; 5 Clinical and Molecular Genetics Unit, Institute of Child Health, University College London, London, UK and Department of Pediatrics, Academic Medical Center, University of Amsterdam, Amsterdam, The Netherlands; 6 Oxford Partnership Comprehensive Biomedical Research Centre, Oxford Radcliffe Hospitals NHS Trust and The University of Oxford, The Wellcome Trust Centre for Human Genetics, Churchill Hospital, Oxford, UK; 7 Department of Genome Sciences, University of Washington School of Medicine and the Howard Hughes Medical Institute, Seattle, Washington, USA

## Abstract

**Background:**

Genomic disorders are often caused by non-allelic homologous recombination between segmental duplications. Chromosome 16 is especially rich in a chromosome-specific low copy repeat, termed LCR16.

**Methods and Results:**

A bacterial artificial chromosome (BAC) array comparative genome hybridisation (CGH) screen of 1027 patients with mental retardation and/or multiple congenital anomalies (MR/MCA) was performed. The BAC array CGH screen identified five patients with deletions and five with apparently reciprocal duplications of 16p13 covering 1.65 Mb, including 15 RefSeq genes. In addition, three atypical rearrangements overlapping or flanking this region were found. Fine mapping by high-resolution oligonucleotide arrays suggests that these deletions and duplications result from non-allelic homologous recombination (NAHR) between distinct LCR16 subunits with >99% sequence identity. Deletions and duplications were either de novo or inherited from unaffected parents. To determine whether these imbalances are associated with the MR/MCA phenotype or whether they might be benign variants, a population of 2014 normal controls was screened. The absence of deletions in the control population showed that 16p13.11 deletions are significantly associated with MR/MCA (p = 0.0048). Despite phenotypic variability, common features were identified: three patients with deletions presented with MR, microcephaly and epilepsy (two of these had also short stature), and two other deletion carriers ascertained prenatally presented with cleft lip and midline defects. In contrast to its previous association with autism, the duplication seems to be a common variant in the population (5/1682, 0.29%).

**Conclusion:**

These findings indicate that deletions inherited from clinically normal parents are likely to be causal for the patients’ phenotype whereas the role of duplications (de novo or inherited) in the phenotype remains uncertain. This difference in knowledge regarding the clinical relevance of the deletion and the duplication causes a paradigm shift in (cyto)genetic counselling.

Non-allelic homologous recombination (NAHR) between neighbouring intrachromosomal segmental duplications (also termed low copy repeats, or LCRs) is the main mechanism underlying genomic disorders. Several recurrent clinical syndromes are caused by either gains or losses of sequences flanked by segmental duplications. Since the initial discovery that Charcot–Marie–Tooth disease and HNPP (hereditary neuropathy with liability to pressure palsies) are caused by the duplication and the reciprocal deletion at 17p11.2, respectively, many other recurrent clinical syndromes have been shown to be caused by NAHR.^1^ The finding that around 4% of the human genome is made up of intrachromosomal segmental duplicons led to speculation that many more unrecognised recurrent rearrangement syndromes might exist.^2^ The advent of array comparative genome hybridisation (CGH) has enabled genome-wide screening for copy number variations (CNVs) in large patient populations leading to the identification of several novel low copy repeat-mediated rearrangements.^3^ ^4^

Chromosome 16 is especially rich in intrachromosomal segmental duplications. During recent primate evolution, chromosome 16 has undergone intense segmental duplication activity, and > 10% of the euchromatic region of the p arm is composed of highly complex low copy repeats.^5^ ^6^ During evolution, these blocks were generated in a stepwise fashion, generating multiple subunits termed LCR16a–t, with a size range of 20–600 kb and sharing >97% sequence similarity.^7^ The complex architecture of chromosome 16p therefore suggests it as an excellent candidate region for novel microdeletion syndromes.^3^ Recently, Ballif *et al*^8^ reported a novel microdeletion syndrome of 16p11.2–p12.2, which seems to be mediated by NAHR between 16p segmental duplicons. In addition to this novel syndrome, various studies analysing CNVs in patients with mental retardation and/or multiple congenital anomalies (MR/MCA) have reported imbalances of 16p13.^9^^–^11 However, the relevance of these findings remains unclear.

During the screening of 1027 patients with MCA and/or MR, we identified 6 deletions and 7 duplications of 16p12–p13, apparently caused by NAHR between LCR16. Our detailed analyses of these imbalances define 16p13.11 as a region of recurrent microdeletion/duplication and suggest that the deletion is a risk factor for MR/MCA but the clinical relevance of the duplication is uncertain.

## METHODS

### Selection of patients

This study involved patients with MR/MCA, ascertained from three sources: (1) those diagnosed by the clinical geneticist of Leuven, Belgium (n = 500), (2) children and young adults from a variety of UK clinical genetics centres, community learning disability teams and other sources, including hospital neuropaediatricians (n = 372),^3^ and (3) DNA from autopsies of fetuses with ⩾1 congenital anomalies at Children’s Hospital and Regional Medical Center (Seattle, WA) after death or elective termination (n = 155).^12^ All were reported to have a normal karyotype at 550 G-band resolution, and in many cases cryptic subtelomeric rearrangements and other specific genetic abnormalities had been excluded.

### Array comparative genome hybridisation

Patients were analysed using two different bacterial artificial chromosome (BAC) array CGH platforms. Patient samples from Leuven, Belgium (n = 500) were hybridised to a custom BAC array with clones spaced at approximately 1 Mb intervals throughout the genome, according to the protocol of Menten *et al*.^13^ Regions were scored as CNVs if one clone passed the threshold of 4× the normal standard deviation (SD), and if ⩾2 flanking clones passed the threshold of log_2_(3/2)−2×SD.^14^ Patient samples from the UK (n = 372) and USA (n = 155) were hybridised to a custom BAC array consisting of ∼2000 clones targeted to regions of the genome flanked by segmental duplications.^10^ Regions were scored as CNVs if the log_2_ ratio of ⩾2 consecutive clones each exceeded 2×SD of the autosomal clones in dye-swap replicate experiments.^3^

An additional patient with a 16p13.11 duplication was identified by array CGH to human 105K genome-wide oligonucleotide arrays (Agilent Technologies, Diegem, Belgium) according to the manufacturer’s instructions. In brief, genomic DNAs from the patient and from a single sex-matched reference patient were separately double-digested using the restriction endonucleases *Alu*I and *Rsa*I (Promega, Leiden, The Netherlands) and purified using Microcon centrifugal filter devices (Millipore Corporation, Missouri, Minneapolis, USA). Then 1.5 μg of the digested products were differentially labelled by random priming with Cy3-dUTP and Cy5-dUTP (Perkin Elmer, Foster City, California, USA) and cohybridised to the array for 48 hrs at 65°C in a rotating oven. Parental DNAs were also hybridised using this method. The hybridised arrays were washed and scanned (Microarray Scanner; Agilent). Image data were extracted using Feature Extraction V.8.5 software (Agilent) and the data analysed using CGH Analytics V.3.4 software (z-score method setting) (Agilent).

To refine thebreakpoints of these rearrangements, we utilised oligonucleotide arrays. One oligoarray consisting of 385 000 isothermal custom made probes (length 45–75 bp) covering a number of chromosomal regions, including this 5 Mb region of chromosome 16p (mean density, 1 probe per 131 bp) (NimbleGen Systems, Madison, Wisconsin, USA). Hybridisations were performed as described previously,^15^ and samples from a single normal man were used as the reference (GM15724; Coriell, Camden, New Jersey, USA).

The second oligoarray used (GeneChip Human Mapping 262K *Nsp*I; Affymetrix, Santa Clara, California, USA) contains 262 264 25 oligonucleotides. In this experimenrt, 250 ng genomic DNA was processed according to the manufacturer’s instructions (Affymetrix GeneChip Human Mapping 500K Manual; http://www.affymetrix.com). Copy number was assessed using DNA-Chip Analyzer (dChip) software 2006.^16^ Regions of copy number gain and loss were found using the hidden Markov model output of dChip.

### Real-time quantitative PCR

Real-time quantitative PCR (qPCR) was performed as previously described^13^ with minor modifications. Primers were designed from RepeatMasked sequence (www.repeatmasker.org/) using Primerexpress V.2.0.0 oligo design software (Applied Biosystems, Lennik, Belgium), and validated with in silico PCR and genome searching using Blat software (http://genome.ucsc.edu/). Single-nucleotide polymorphisms (SNPs) were excluded from the primer sequence (SNP track in UCSC Browser). Real-time qPCR was performed using a commercial preparation (Q-PCR MasterMix Plus for SYBR Green I without UNG (uracil-*N*-glycosylase); Eurogentec, Liege, Belgium) according to the manufacturer’s instructions. Each reaction was performed in duplicate in a final volume of 15 μl containing 6–30 ng/μl genomic DNA, 1.25 μM forward/reverse primer, and 7.5 μl SYBR Green mastermix.

### Control populations

The first control population comprised 722 unrelated individuals from Belgium who had been referred for clinical genetic testing for haemochromatosis or cystic fibrosis. Genomic DNA of each individual was extracted from blood lymphocytes according to standard procedures, and assayed by real-time qPCR using primer pair 3 (Eurogentec, Seraing, Belgium) for detection of copy number changes of 16p13.1. Thresholds were set at a fold difference of 0.8 and 1.3. All samples surpassing these thresholds were analysed twice to confirm the presence of copy number changes.

A second control population, comprising 960 unrelated Caucasian adults (age 40–70 years) from the USA, were genotyped using a chip array (HumanHap300 Genotyping BeadChips; Illumina, San Diego, California), comprising ∼317 000 HapMap SNPs distributed throughout the genome. Each individual was enrolled in the Pharmacogenomics and Risk of Cardiovascular Disease (PARC) study, which aims to identify genetic contributors to the variable efficacy of statin drugs on cardiovascular disease risk (http://www.pharmgkb.org/do/serve?objId = 5&objCIs = Project). Hybridisations, data analysis and copy number analysis, focused on this region of 16p, were performed according to published protocols.^17^

## CASE REPORTS

### Patients carrying 1.65 Mb 16p13.11 deletions

#### Patient 1 (ID 224725)

This adult patient is the only affected member of five siblings. She has severe MR, therapy-resistant epilepsy and behavioural problems. She has short stature (143 cm, less than third percentile (P3) of 155 cm) and microcephaly (occipitofrontal circumference (OFC) 51 cm; P3  = 52.2 cm). She has a short nose, smooth philtrum, wide mouth and fine palpebral fissures. She has difficulty in expressive language and has an ataxic gait.

#### Patient 2 (ID 335606)

This adult proband and his brother were referred with severe MR. The proband has short stature (150 cm; P3  = 168 cm), microcephaly (OFC 51 cm; P3  = 52.2 cm), pectus excavatum and limb spasticity. He is being treated for epilepsy.

#### Patient 3 (ID 22698)

This adult man with moderate MR is the only child of non-consanguineous parents. His stature and OFC are normal and he is obese (103 kg; >P97). He is not dysmorphic, is extremely talkative, and displays intermittent verbal aggression and self-mutilation.

#### Patient 4 (ID 224358)

This patient is the male sibling of a dizygotic twin pregnancy that occurred via in vitro fertilisation with intracytoplasmic sperm injection (ICSI). At 16 weeks gestation, ultrasound examination demonstrated abnormalities in the female fetus. The pregnancy went to term. The male fetus has a ventricular septal defect (VSD) and a right-sided aorta. He is subject to frequent infections because of his low CD3 count – he has a 22q11 deletion. The 1856 g cyclopic female fetus had a crown rump length of 28.5 cm and a head circumference of 26 cm (proportionally microcephalic). Holoprosencephaly, agenesis of the nose, a midline upper lip notch and a median cleft palate were present together with two pre-auricular tags on the left, and a right dysplastic ear with a pre-auricular tag and an atretic auditory canal.

#### Patient 5 (ID FA-180)

Prenatal ultrasound at 18 weeks gestation showed a cleft lip on the right, a possible intracranial abnormality with dilated lateral ventricles, and thinning of the cortical mantle. The pregnancy was terminated at 21 weeks. Autopsy showed post-haemorrhagic hydrocephalus with marked ventriculomegaly, cortical thinning, a hypoplastic falx cerebri, cleft lip on the right side, two preauricular skin tags on the right, and cleft T1 and T3 vertebral bodies. Physical growth parameters were consistent with gestational age. This patient was reported by Mefford *et al.*^12^

### Patients carrying 1.65 Mb 16p13.11 duplications

#### Patient 6 (ID 325275)

The second child of non-consanguineous parents, this patient was born after an uneventful pregnancy. He has moderate MR. He was constipated from birth and at 6 months was diagnosed with Hirschsprung disease, for which he had a resection and an end-to-end anastomosis. Behavioural problems developed at 5 years of age and he has received special education. As an adult, he has normal biometry, bushy eyebrows and a mandibular overbite.

#### Patient 7 (ID IMR277)

This patient was originally reported by Kriek *et al*^9^ as part of a multiplex ligation-dependent probe amplification (MLPA) screen of 105 patients selected for developmental delay and/or congenital malformations. He had learning problems in primary school, but is not clearly dysmorphic and has no serious behavioural problems. The extent of the duplication was verified using array CGH and fluorescent in situ hybridisation. MLPA and real-time qPCR of the parents confirmed the de novo nature and the size of this duplication.

#### Patient 8 (ID IMR277)

This patient has severe learning disabilities with limited use of language, poor vocabulary and repetitive speech. She displays challenging, agitated behaviour marked by shouting, hand-clapping, kicking, hitting and throwing objects at people, although this has improved with age. She has epilepsy.

#### Patient 9 (ID IMR220)

The patient is the only child of non-consanguineous parents. He walked at 13 months, and later was described as clumsy but not dyspraxic. Speech has always been problematic. His delay was moderate (IQ of 55), and he had behavioural problems such as increased impulsivity and limited attention control. Grommets were inserted for upper airway infections, but otherwise there were no major physical problems. At 14 ¾ years of age, OFC was 54.7 cm (50th centile). He was noted to have large simple ears, thick lips, a large tongue, and large and somewhat puffy hands with small nails. CT scan of the brain was normal. Metabolic screening of urine showed no abnormalities. EEG was normal. Ophthalmological examination showed bilateral astigmatism. Fragile X syndrome (FRAXA) testing showed a full expansion in the patient and a premutation in his mother, which had been inherited from her father.

#### Patient 10 (ID OxP078)

An ultrasonography examination at 20 weeks gestation revealed microcephaly in this patient. This was confirmed after delivery and at 4 years of age the OFC was 44 cm (P3 = 48.6 cm). The patient had brachycephaly, telecanthus, abnormal eyebrows, deep-set eyes, epicanthic folds, a pinched nasal tip, prominent nose and small jaw. She also had a VSD, an umbilical hernia, deep creases on the palms and soles, and clinodactyly. An MRI scan showed a small brain with delayed myelination and prominent extra axial fluid-filled spaces. The patient has delayed speech and hyperactive behaviour with aggressive episodes. At 6 years of age, she attends normal school with a large amount of learning support. The child’s mother also has a small head, mild learning difficulties and poor temper control. Her mother (the proband’s maternal grandmother) has a small head and mild learning difficulties. One developmentally and behaviourally normal maternal aunt carries the duplication and one aunt with mild learning difficulties and aggressive behavioural is cytogenetically normal.

### Patients carrying larger atypical 16p13.11 rearrangements

#### Patient 11 (ID 66529)

The only child of non-consanguineous parents, this patient was born at term with a birth weight of 3200 g. She had feeding and respiratory difficulties in the neonatal period. As an adult , she has an asymmetrical face with left facial nerve paresis, a short neck with reduced mobility, bilateral epicanthal folds, strabismus, choroid colobomata, and previously had atresia of the right choana. An atrial septal defect type 2 closed spontaneously and she has a unique right kidney with double ureters. She has hearing difficulties, and a CT scan showed aplasia of the semicircular canals and abnormal middle ear bones. Her behaviour is normal. *CHD7* mutation analysis was normal.

#### Patient 12 (ID IMR184)

This patient was reported in Sharp *et al.*^3^

#### Patient 13 (ID 20754)

This sixth child, born to a mother with pre-eclampsia, weighed 1800 g at full term. She had neonatal seizures and marked developmental delay, not walking and talking until 4 years of age. As an adult, she has has short stature (150 cm; P3 = 155 cm) and microcephaly (OFC 50.5 cm; P3 = 52.2 cm). She has an IQ of 38 and has cyclic depression.

## RESULTS

### Recurrent microdeletion/duplication of 16p13.11

**Figure 1:**
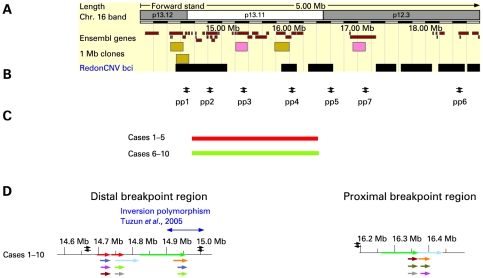
Molecular overview of recurrent deletions and reciprocal duplications in patients 1–10. (A) ENSEMBL overview (freeze: 24-04-2007) which visualises 1 Mb bacterial artificial chromosome (BAC) clones, the genes involved in the imbalance, the copy number variable regions^19^ and chromosome bands. (B) The location of real-time quantitative PCR primers used to fine-map the breakpoints, are depicted. (C) The extent of the deletion and duplication is shown by red and green bars, respectively in typical del/dup patients. (D) Organisation of the segmental duplication structure at the distal and proximal breakpoints of recurrent 16p13.1 rearrangements. Each coloured bar represents a pairwise alignment with >98% identity.

**Figure 2:**
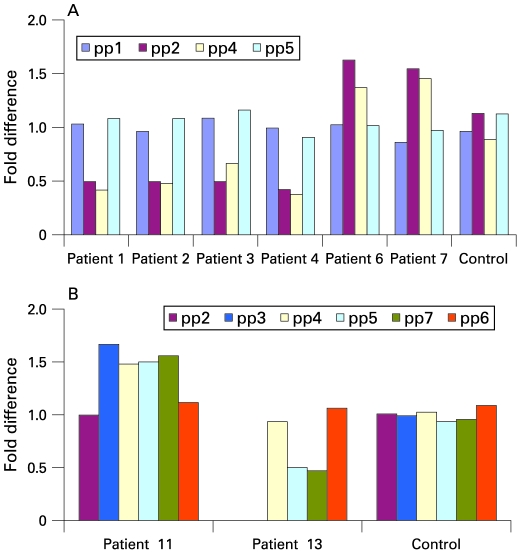
Real-time quantitative PCR results with different primer sets flanking the breakpoint regions. (A) In all individuals carrying the common deletion/duplication (patients 1–10), the distal breakpoints occur between primers pp1 and pp2, and the proximal breakpoint is defined by primers pp4 and pp5. (B) The extent of the atypical duplication and deletion identified in patients 11 and 13 were delineated by primersets pp2/pp3 and pp4/pp5, respectively, for distal breakpoints and between pp6 and pp7 for the proximal breakpoint. The presence of two copies for a locus was defined by a fold difference of 1 whereas a fold difference of 0.5 or 1.5 corresponds to a deletion or a duplication, respectively.

Array CGH was performed on 1027 patients or fetuses with unexplained MR/MCA using one of two custom BAC arrays.^10^ ^13^ In total, 13 patients with 16p12–p13 rearrangements were found (1.3%), 6 of whom carried a deletion and 7 a duplication. Ten of these rearrangements (five deletions and five duplications, patients 1–10) seemed to involve the same ∼1.65 Mb region of 16p13.11, suggesting that they probably represent the reciprocal events of NAHR (fig 1). Three additional patients carried atypical larger rearrangements. These were up to 3.4 Mb in size, and either overlapped or flanked the more common 1.65 Mb 16p13.11 deletions/duplications seen in the other 10 patients. Real-time qPCR was used to confirm the results of the array CGH analysis, and clearly distinguished patients with deletions and duplications from normal controls (fig 2). For one patient with a typical deletion (patient 4), testing of parental DNA showed that the microdeletion was inherited from the phenotypically normal father. In contrast, for one patient with a typical duplication (patient 10), testing revealed three generations in which the duplication initially seemed to segregate with a mild or more severely abnormal phenotype, but testing of additional family members showed that the duplication and phenotype did not cosegregate.

### Rearrangement breakpoints localise to LCR16 segmental duplication blocks containing a positively selected gene family

**Figure 3:**
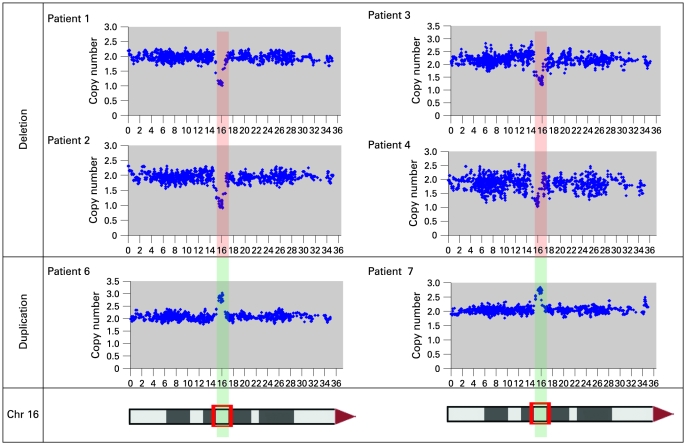
Detection of 16p12–p13 imbalances defined by the 262k *NspI* SNP array. Data from the common 16p13.11 1.65 Mb rearrangements are shown. Each plot has physical probe position on 16p (*x*-axis) against probe intensity ratio (*y*-axis). Red shading, common deleted region; green shading, duplication in patients 1–4, 6 and 7. Chr, chromosome.

**Figure 4:**
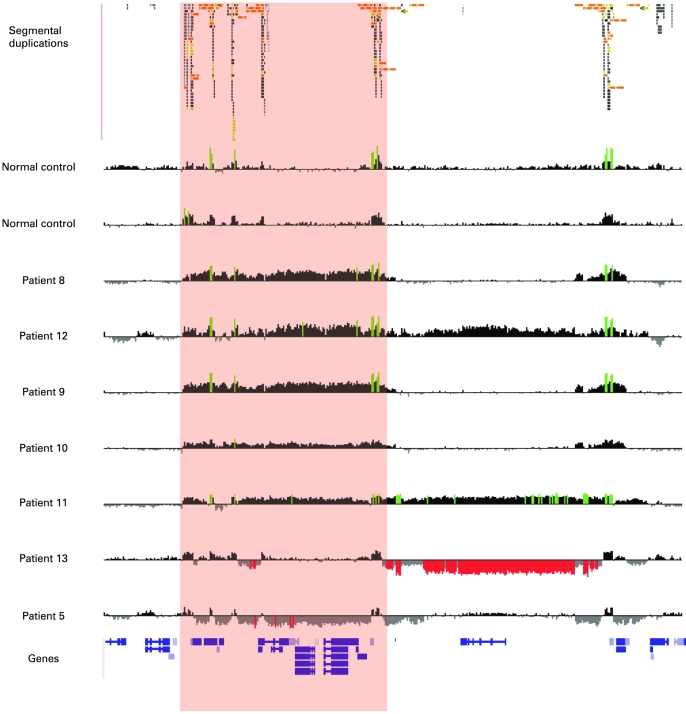
High-resolution oligonucleotide array mapping of seven 16p12.3–p13.11 rearrangements, of which four have a common distal breakpoint (14.7–14.75 Mb). For those four patients with the common 1.65 Mb rearrangement (red shading), the proximal breakpoints also map to a second LCR16 cluster (16.3–16.77 Mb). Another three patients have an atypical rearrangement: patients 11 and 12 show an atypical larger duplication, with the distal breakpoint between 15.0–15.4 Mb and the proximal breakpoint located within a third LCR16 cluster (18.3–18.4 Mb), and patient 13 has an atypical deletion with proximal breakpoint in the third LCR16 cluster and distal breakpoint in the second cluster. Data from normal control individuals show that there is marked copy number variation in the LCR16 clusters that define these three breakpoint regions. Note that the high degree of homology between these LCR16s also results in false-positive signals from probes that are identical to those within the true deletion/duplication in patients 5, 8 and 9. The image has a 5 Mb region of 16p12–p13 (chr16:14 000 000–19 000 000). For each individual, deviations of probe log_2_ ratios from zero are depicted by grey/black lines, with those exceeding a threshold of 1.5 SD from the mean probe ratio shown in green and red to represent relative gains and losses, respectively. Segmental duplications of increasing similarity (90–98%, 98–99%, and >99%) are represented by grey/yellow/orange bars, respectively.

Each rearrangement was mapped at higher resolution using either 262k *NspI* SNP arrays (Affymetrix) (fig 3) or a custom oligonucleotide array (Roche Nimblegen, Madison, Wisconsin, USA) (fig 4). These data confirmed the results of the BAC array CGH, and localised the breakpoints of each rearrangement to segmental duplications composed of large clusters of LCR16.^7^ Significantly, patients 1–10 had both proximal and distal breakpoints that localised to the same intervals (distal breakpoints, 14.7–14.75 Mb, proximal breakpoints 16.3–16.77 Mb). These duplication blocks, which define the common breakpoint regions, were also found to be strongly polymorphic in copy number in normal controls (fig. 4, supplementary material online). These polymorphic breakpoint regions correspond to LCR16a/LCR16b motifs, which have been shown to have undergone rapid proliferation during primate evolution and contain a gene family, *Morpheus*, which has a signature of extreme positive selection.^18^ Real-time qPCR using primers flanking these segmental duplications confirmed that that the breakpoints occurred within these LCRs (fig 2).

There are 15 RefSeq genes in the common 1.65 Mb recurrent rearrangement region. Given that microcephaly and brain malformations are the major recurrent characteristic in our patients with deletions, *NDE1* was believed to be a good candidate for a dosage-sensitive gene that might underlie the features of these deletions. In order to test the hypothesis that the deletion acts by unmasking the presence of a recessive mutation on the remaining allele, we sequenced the *NDE1* gene in four of our patients with deletions (1–4). Primers covering all eight exons and the 5′ and 3′ untranslated regions (suppplementary table 1 online) were used for direct sequencing of genomic DNA, but no mutations were found.

### The microdeletion associates with the MR/MCA phenotype

Although neither 16p13.11 duplications or deletions have been found in 210 unrelated HapMap individuals,^19^ nor in 122 other individuals sampled from the normal population,^10^ ^20^ ^21^ a larger and population matched sample size was needed in order to draw a statistically meaningful conclusion as to their pathogenic significance. Therefore, the copy number of the commonly rearranged 16p13.1 region in 722 population matched controls ascertained from the Belgian population, and in a further 960 Caucasian controls drawn from the USA, were evaluated. These analyses did not detect any deletions of this region in the 1682 controls tested, but did reveal the presence of five 16p13.11 duplications. Although we do not have access to detailed clinical information for these five controls, the method used to ascertain them suggests that they are unlikely to have significant developmental abnormalities, but we cannot exclude possibility that they might have abnormal learning, memory or behavioural characteristics. Combining these data with previously published analysis of 332 controls results in a total control population of 2014, including 5 carriers of 16p13.1 duplications, but no carriers of deletions. This compares with 5 deletions and 7 duplications ascertained from 1027 patients with MR/MCA. We therefore conclude that deletions of 16p13.1 are significantly associated with patient phenotype (p = 0.0048, Pearson χ^2^ test with simulated p value, based on 10 000 replicas), but show incomplete penetrance, as demonstrated by their presence in some apparently unaffected relatives. The incidence of duplications in association with disease is not significantly different from that in controls (p = 0.1273, Pearson’s χ^2^ test). This observation leads us to a number of possible conclusions regarding the duplication including that it might (1) be truly clinically benign, (2) be compatible with a phenotype that has passed unnoticed (or has not been excluded) in the control population tested or (3) work in combination with other predisposing factors to give an MR phenotype.

### Atypical rearrangements of chromosome 16p12–p13

In addition to the common recurrent 1.65 Mb microdeletion/duplication of 16p13.11 observed in 10 patients, 3 atypical rearrangements were found. Two patients (11 and 12) carried a larger duplication of ∼3.4 Mb in size, overlapping the typical duplication. The distal breakpoint of these atypical duplications was located between 15.0–15.4 Mb and the proximal breakpoint was located within a third LCR16 cluster (18.3–18.4 Mb). Results of real-time qPCR with primer pair 2 (supplementary table 2 online) were normal whereas the results using primer pair 3 showed the presence of a duplication (fig 2). This result confirms that the duplication starts in a more proximal LCR16 than the typical duplication. This duplicated region includes 12 RefSeq genes. The normal mother of patient 11 was found to be a carrier of this same duplication.

One further patient (patient 13) carried a 1.6–2.1 Mb deletion that flanked the common rearrangement region. This atypical deletion had its distal breakpoint in the second cluster and its proximal breakpoint in the third LCR16 cluster. The region includes 2 RefSeq genes. This imbalance was inherited from a phenotypically normal mother. A summary of all 13 rearrangements found is shown in table 1.

**Table 1:** Genotype–phenotype correlation of patients with deletion or duplication of 16p12–p13

Patient (Deciphercode)	Phenotype	Pattern of inheritance	Type of imbalance	Distal breakpoint (Mb)	Proximal breakpoint (Mb)
1 (CHG00002371)	Severe MR, IQ = 38, short stature (143 cm), microcephaly (51 cm), epilepsy, ataxia	Parents not available for testing	Deletion	14.7–14.75	16.3–16.77
2 (CHG00002372)	Severe MR, short stature (150 cm), microcephaly (51 cm), epilepsy, pectus excavatum, limb spasticity	Affected brother does not carry deletion		14.7–14.75	16.3–16.77
		Parents not available for testing			
3 (CHG00002374–)	Moderate MR, normal stature (183 cm), normal head circumference (55,3 cm), behavioural problems	Parents not available for testing		14.7–14.75	16.3–16.77
4 (CHG00001230)	Term fetus; autopsy showed holoprosencephaly, nose agenesis, midline upper lip notch, midline cleft palate, dysplastic external ear and atretic auditory canal on right, preauricular skin tags bilaterally, relative microcephaly	Phenotypically normal father carries deletion		14.7–14.75	16.3–16.77
5	Fetus at 21 weeks; autopsy showed post-hemorrhagic hydrocephalus with marked ventriculomegaly, cortical thinning, hypoplastic falx cerebri, cleft lip on right, two preauricular skin tags on right, and cleft T1 and T3 vertebral bodies. Physical growth parameters were consistent with gestational age	Parents not available for testing		14.7–14.75	16.3–16.77
6 (CHG00001046)	Moderate MR, normal stature (176 cm), normal head circumference (57.4 cm), behavioural problems, Hirschsprung disease	Mother does not carry duplication	Duplication	14.7–14.75	16.3–16.77
		Father unavailable for testing			
7 (LEI)00002370)	MR, mild developmental delay, learning disabilities. Originally reported by Kriek *et al*.^9^	de novo imbalance		14.7–14.75	16.3–16.77
8	Severe learning disabilities with limited use of language, poor vocabulary and repetitive speech, epilepsy. Challenging, agitated behaviour marked by shouting, hand-clapping, kicking, hitting and throwing objects at people, although this has improved with age	Phenotypically normal father carries duplication		14.7–14.75	16.3–16.77
9	Moderate developmental delay, behavioural problems (increased impulsivity, limited attention span). Large simple ears, thick lips, large tongue, large puffy hands and small nails. Also has an expansion of the *FRAXA* triplet repeat	Parents not available for testing		14.7–14.75	16.3–16.77
10	Microcephaly found at 20 weeks gestation. At 4 years of age OFC 44 cm (P3 = 48.6 cm). Brachycephaly, telecanthus, abnormal eyebrows, deep set eyes, epicanthic folds, pinched nasal tip, prominent nose, small jaw, VSD, umbilical hernia, deep palmar and plantar creases, speech delay, hyperactive behavioural with aggressive episodes. Mother has small head (no OFC)	Mother with mild phenotype carries the duplication		14.7–14.75	16.3–16.77
11 (CHG00000993)	Feeding and respiratory problems as neonate. Asymmetric face with left facial nerve paresis, short neck with reduced mobility, bilateral epicanthal folds, strabismus, choroid colobomata, atresia of right choana, ASDII, unique right kidney with double ureters, aplasia of semicircular canals, abnormal middle ear bones	Phenotypically normal mother carries duplication	Duplication	15.1–15.4	18.05–18.45
12	MR, multiple congenital anomalies. Originally reported by Sharp *et al.*^10^	Parents not available for testing		15.1–15.4	18.3–18.5
13 (CHG00002373)	Marked developmental delay, IQ = 38, short stature (150 cm), microcephaly (50,5 cm), neonatal seizures	Phenotypically normal mother carries deletion	Deletion	16.3–16.77	18.3–18.4

MR, mental retardation; OFC, occiptofrontal circumference.

## DISCUSSION

We describe five patients carrying identical 1.65 Mb deletions of 16p13.11 encompassing 15 genes. In addition, we found five patients carrying apparently reciprocal duplications of this same region. The rearrangement breakpoints are located in low copy repeats implying that non-allelic homologous recombination between these flanking LCRs mediates these rearrangements. Three further patients presenting with larger atypical rearrangements showed breakpoints that also mapped to clusters of LCR16.

Despite careful parental analysis, observations of rearrangements of 16p13.11 make it difficult to distinguish between disease-causing events (generally presumed to be de novo) and benign variants (that do not contribute to a phenotype). Two deletions and one duplication were found to be inherited from an apparently normal parent, and another duplication was inherited from a clinically mildly abnormal parent. Conversely, we also found a duplication that occurred de novo. Previous studies have reported 16p13.11 deletions occurring both de novo and by inheritance from a normal parent, while duplications were reported to be inherited from normal parents in two families.^11^ ^22^ ^23^ One interpretation of these observations could be that rearrangements of this region are benign variants, and that the observed phenotypes are coincidental with the presence of the imbalance. However, one case–control study reported a significantly higher incidence of the del16p13.11 in patients with MR/MCA, implying that the del16p13.11 is a risk factor contributing to the MR/MCA phenotype. The duplication is present in equal frequency in the normal and the patient population, indicating this variant is compatible with a normal phenotype. The identification of this dup16p13.11 in a patient with fragile X (patient 9) in our study lends support to this hypothesis. Recently, Ullmann *et al*^11^ reported three carriers of dup16p13.11 in a cohort of 182 people with autism. Because the duplication was found in 5 out of 2014 normal participants in our study, the duplication seems overrepresented in the autistic population (p = 0.023, Fisher exact test). More studies are needed strengthen this association.

Three of the four adult patients with 16p13.11 deletions had both microcephaly and seizures, and the two fetuses with the deletions had brain anomalies, one of which also had relative microcephaly. The same three adult patients also have small stature. Small stature and dysmorphic features were also a feature of one of the patients described by Ullman *et al*.^11^ In contrast, our fourth adult patient and a previously described deletion carrier^22^ had normal head circumference (55.3 cm), while one previously reported patient had macrocephaly.^11^

Although the typical 16p13.11 duplication may be a benign variant, it is striking that four of our duplication carriers not only presented with MR but also behavioural problems. Similarly, members of two of three families with duplications reported by Ullman *et al*^11^ had similar behavioural problems. Although we do not have data on the behavioural phenotypes of all patients with MR/MCA tested using the arrays, the occurrence of this type of behaviour in this patient population seems higher than average, and hence, it seems plausible that dup16p13.11 carriers may have a predisposition for aggressive behaviour.

To date, most genomic imbalances have been classified as either benign or pathogenic and most microdeletion syndromes are presumed to be well-defined clinical conditions. However, even well-known genomic disorders can be phenotypically heterogeneous and more variable than originally thought, owing to incomplete penetrance or variable expression. The variability of the del22q11 phenotype originally led to their different clinical classification as DiGeorge syndrome (heart and thymus defects) (OMIM 188400), Sprintzen syndrome (speech difficulties) or velocardiofacial syndrome (VCFS) (conotruncal anomaly face) (OMIM 192430). Recently, several reports have been published about atypical patients with 22q11 deletions.^24^ ^25^ Equally, in dup22q11 carriers, the phenotype may range from severe MR through to completely unaffected, and various minor developmental anomalies are noted.^26^ ^27^ In addition, 22q11 duplications can be inherited from apparently normal parents.^26^ ^28^ Therefore, it is possible that 16p13.11 duplications might also be causative and the heterogeneous phenotype of our patients explained in part by (1) the unbiased selection criteria, (2) variability due to other genetic or possibly environmental determinants, (3) incomplete penetrance, (4) variable expression or (5) unmasking of recessive alleles.^29^ In particular, owing to the presence of del16p13.11 in a normal parent of one of our probands, we set out to test the latter hypothesis. Because microcephaly was observed in two of the three adult patients with typical deletions and in one of the two fetuses with deletion, *NDE1* was considered an excellent candidate gene for this phenotype. *NDE1* is strongly expressed in brain,^30^ and forms complexes with *LIS1*, a dosage-sensitive gene that is crucial for neuronal migration and cerebral development, and that is known to underlie Miller–Dieker lissencephaly syndrome (OMIM 247200). Furthermore, *Nde1*-null mice show microcephaly.^31^ However, sequencing of all exons of *NDE1* in four patients with deletions did not reveal any mutation on the remaining allele, suggesting that this is not the mechanism responsible for the phenotype in our patients. Another plausible candidate gene is *NTAN1* (asparagine-specific N-terminal amidase). Mouse models deficient for this enzyme showed alterations in activity, social behaviour and memory.^32^ However although this region is reported in the Redon database (http://projects.tcag.ca/variation/) to have CNVs, learning and memory defects and aberrant social behaviour could not be excluded among controls and therefore the *NTAN1* gene remains a good candidate gene.

Imprinting is a mechanism that could potentially explain the presence of these rearrangements in unaffected relatives.^11^ However, there are no known imprinted genes on chromosome 16 (http://www.geneimprint.com/site/genes-by-species). Furthermore, for both deletions and duplications of 16p we observed the inheritance of these imbalances from normal parents through both the maternal and paternal germ lines, indicating that imprinting does not significantly influence patient phenotype of this disorder.

In addition to the common 1.65 Mb rearrangement observed in the 10 patients reported here, 3 “atypical” chromosomal imbalances either overlapping or flanking this common region were found in patients with MR/MCA. Breakpoints for all three imbalances were also located within LCR16 sequences. However, the atypical 3.4 Mb duplication seems to be mediated by different LCR16s compared with the atypical deletion. Therefore, the complex structure of the LCR16s in this region seems to be involved in generating a variety of different chromosomal rearrangements. The finding of variably sized rearrangements on chromosome 16p is similar to that observed for other recurrent genomic disorders, such as the Prader–Willi/Angelman syndrome,^33^ Smith–Magenis syndrome,^34^ and the 15q24 deletion syndrome,^35^ in which recombination within alternate LCRs can result in recurrent deletions and duplications of different sizes.

In conclusion, we report a novel genomic disorder likely caused by NAHR between copies of LCR16. Although in some cases this is inherited from a normal parent, we found a strong association of the deletion with developmental disorders. Reciprocal duplications were observed as both inherited and de novo events, and were also identified in several controls, suggesting that the duplication by itself confers either no phenotype at all or a range of phenotypes of varying severity. Alternatively, the duplication may require additional predisposing factors to have a phenotypic effect. Our findings have important implications for genetic counselling. Traditionally, chromosomal imbalances inherited from a normal parent were considered benign, while de novo chromosomal imbalances were considered pathogenic. Although our results suggest that the inherited 16p13.11 deletion is likely causal for the phenotype, the clinical significance of both de novo and inherited duplications remains uncertain and they may be benign variants. The study of additional patients and normal individuals with 16p13.11 rearrangements is required to reinforce this hypothesis and to obtain better insight in the potential pathology associated with the observed microdeletion and microduplication events.
